# Approaches to isolation and molecular characterization of disseminated tumor cells

**DOI:** 10.18632/oncotarget.5568

**Published:** 2015-09-10

**Authors:** Mark Jesus M. Magbanua, Rishi Das, Prithi Polavarapu, John W. Park

**Affiliations:** ^1^ Division of Hematology/Oncology, Helen Diller Family Comprehensive Cancer Center, University of California San Francisco, San Francisco, CA, USA

**Keywords:** disseminated tumor cells, disseminated cancer cells, micrometastatic cells, micrometastasis, minimal residual disease

## Abstract

Micrometastatic cells in the bone marrow, now usually referred to as “disseminated tumor cells (DTCs)”, can be detected in early stage cancer patients. It has been hypothesized that DTCs represent key intermediates in the metastatic process as possible precursors of bone and visceral metastases, and are indicators of metastatic potential. Indeed, multiple clinical studies have unequivocally demonstrated the prognostic value of these cells in breast and other cancers, as DTCs have been associated with adverse outcomes, including inferior overall and disease-free survival. Despite this established clinical significance, the molecular nature of DTCs remains elusive. The complexity of the bone marrow poses a unique challenge in the isolation and direct characterization of these rare cells. However, recent advances in rare-cell technology along with technical improvements in analyzing limited cell inputs have enabled the molecular profiling of DTCs. In this review, we discuss research featuring the isolation and genomic analysis of DTCs. Emerging work on the molecular characterization of DTCs is now providing new insights into the biology of these cells.

## INTRODUCTION

### Background

The lethal progression of solid tumors usually occurs via hematogenous spread from the primary tumor to distant sites. One of the potential steps in this metastatic process involves tumor cells— termed micrometastases or disseminated tumor cells (DTCs)— transiting from the primary tumor to the bone marrow. Current detection methods for DTCs include immunocytochemical (ICC) assays as well as nucleic acid-based assays using reverse transcriptase-polymerase chain reaction [1, 2]. A number of studies have shown that ICC-based detection of DTCs is a prognostic factor indicating adverse disease-free and overall survival in patients with early stage cancers [3-10]. The detection of DTCs, however, has yet to become a standard component of disease staging, partly due to the lack of standardized approaches for phenotypic identification of these cells (see “Methodological Issues for DTC Detection”).

### Rationale for DTC profiling

Despite the prognostic significance of DTCs, their biology is not well understood. It has been hypothesized that DTCs hold particular significance in cancer metastasis, including: as potential precursors of overt bone or bone marrow metastasis, as a reservoir of cells for further circulation and metastasis to visceral sites, and as a prognostic factor of metastatic potential. Because of their rarity (1 per 10^6^-10^7^ bone marrow cells) and the inherent complexity of the bone marrow compartment, major technical challenges have impeded the detection and isolation of DTCs. However, novel strategies for molecular profiling of DTCs have recently emerged, and will potentially shed new light on these rare and elusive cells.

DTC profiling may provide insights into the process of metastasis, including key aspects regarding genomic instability, tumor heterogeneity, tumor dormancy, cancer stem cells and epithelial-to-mesenchymal transition. In addition to pathogenetic insights, results from DTC profiling could in principle be used as new biomarkers in the clinical management of early cancers. The established prognostic significance of DTCs at time of diagnosis may be improved by the incorporation of additional phenotypic information about the true metastatic potential of these cells, leading to more accurate risk stratification for treatment decision-making. In addition to initial testing at time of diagnosis, DTC sampling and profiling can be performed before, during and after neoadjuvant or adjuvant therapy. DTC profiling in these settings may enable more rational treatment selection, early identification of failing treatments, and discovery of disease evolution requiring alternative treatments.

### Literature review methods

In this paper, we reviewed scientific literature describing efforts on genomic and transcriptional profiling of DTCs. We performed a PubMed search using the search terms, “disseminated tumor cells”, “disseminated cancer cells”, and “micrometastatic cells” and identified 400 relevant articles published within the last two decades (up to December 2014) (Supplementary Figure 1). Reference lists in selected publications were also crosschecked to identify additional related papers. Abstracts were then reviewed to include studies involving multigene quantitative polymerase chain reaction (QPCR) analysis and other genome-wide approaches including comparative genomic hybridization (CGH), gene expression microarray, and next generation sequencing (NGS) analyses. We found 18 original articles that fit our search criteria and are listed in Table 1.

**Table 1:** Studies on genomic profiling of disseminated tumor cells

Study	Type of cancer	Stage	Type of molecular analysis	Molecular assay utilized	Input for molecular analysis	Matched Tumors Analyzed	Evidence for Clonal or Parallel Evolution
Klein et al, 1999 PNAS	CUP	Metastatic	Copy number, LOH, Mutation screen	cCGH, Microsatellite analysis, PCR-RFLP, Sanger sequencing	Isolated single cells	Metastatic lesion (liver)	n.a.
Klein et, 2002 Lancet	B,P and GI	Non-metastatic and metastatic	Copy number, Mutation screening	cCGH, Single-stranded conformational polymorphism analysis, Sanger sequencing	Isolated single cells	Lymph node	Parallel
Klein et al, 2002 Nat Biotechnol	C,L and B	Non metastatic and metastatic	Gene expression, Copy number	cCGH, Dot-blot hybridization	Isolated single cells	None	n.a.
Schmidt-Kittler et al, 2003 PNAS	B	Non-metastatic and metastatic	Copy Number, LOH	cCGH, Microsatellite analysis	Isolated single cells	Primary tumor and lymph node	Parallel
Kraus et al, 2003 Genes Chromosomes Cancer	P	Non-metastatic	Copy Number	cCGH, aCGH, M-FISH	Bulk cultured cells	Primary tumor	Clonal
Gangnus et al, 2004 Clin Cancer Res	B	Non-metastatic	Copy number	cCGH	Isolated single cultured cells	Primary tumor	Parallel
Schardt et al, 2005 Cancer Cell	B	Non-metastatic and metastatic	Copy Number, LOH	cCGH, Microsatellite analysis, PCR-RFLP, OPCR	Isolated single cells	Primary tumor	Parallel
Watson et al, 2007 Clin Cancer Res	B	Non-metastatic	Gene Expression	Expression microarray, QPCR	Enriched bone marrow	None	n.a.
Fuhrmann et al, 2008 Nucleic Acids Res	B	n.d.	Copy Number	cCGH, aCGH, QPCR	Isolated single cells	None	n.a.
Stoecklein et al, 2008 Cancer Cell	E	Non-metastatic and metastatic	Copy Number	cCGH, QPCR	Isolated Single cells	Primary tumor	Parallel
Holcomb et al, 2008 Cancer Res	P	Non-metastatic and metastatic	Copy number	aCGH	Pooled cells (10–20)	Primary tumor	Parallel
Weckermann et al, 2009 J Clin Oncol	P	Non-metastatic and metastatic	Copy number	cCGH	Isolated single cells	Primary tumor	Parallel
Mathiesen et al, 2012 Int J Cancer	B	Non-metastatic and metastatic	Copy Number	aCGH	Isolated single cells	Primary tumor	Clonal
Siddappa et al, 2012 Breast Cancer Res Treat	B	Non-metastatic	Gene Expression	Digital molecular barcoding, QPCR	Enriched bone marrow	None	n.a.
Moller et al, 2013 Front Oncol	B	Non-metastatic	Copy Number, Copy neutral LOH	aCGH, Next generation sequencing	Isolated single cells	Primary tumor	Clonal
Czyz et al, 2014 PLoS One	B	Metastatic	Copy Number	aCGH	Isolated single cells	Primary tumor and lymph node	Parallel
Chery et al, 2014 Oncotarget	P	Non-metastatic and metastatic	Gene Expression	Expression microarray	Isolated single cells	None	n.a.
Guzvic et al, 2014 Cancer Res	P	Non-metastatic and metastatic	Gene expression, Copy number	PCR, cCGH, aCGH	Isolated single cells	None	n.a.

Abbreviations: CUP-cancer of unknown primary, B-breast, P-prostate, GI-gastrointestinal tract, C-cervical, L-lung, E-esophageal, LOH-loss of heterozygosity, cCGH-chromosome comparative genomic hybridization, PCR-RFLP- polymerase chain reaction-restriction fragment length polymorphism, aCGH-array comparative genomic hybridization, M-FISHmulticolor fluorescence in situ hybridization, QPCR-quantitative polymerase chain reaction.

### Methodological issues for DTC detection

Unfortunately, efforts to implement clinical detection of DTCs have been beset by multiple methodological issues [11, 12]. The wide variety of ICC reagents and strategies can clearly impact DTC detection results. For example, comparison of different anti-cytokeratin antibodies for DTC detection showed significant variability in detection rates [13]. Additionally, some methods may preferentially detect certain tumor phenotypes. As such, greater effort in standardization and head-to-head comparisons of DTC enumeration assays is warranted prior to routine adoption in the clinic. DTCs identified by candidate approaches should be subjected to detailed characterization to establish proof of malignant origin.

In contrast to DTC research, circulating tumor cell (CTC) detection in peripheral blood illustrates successful translation to commercialization and subsequent clearance by the U.S. Food and Drug Administration. The CellSearch^®^ system has allowed for the reliable and reproducible enumeration of CTCs. Clinical studies in metastatic breast, prostate and colon cancer patients have shown that elevated CTC numbers correlate with poor survival [14-16]. Other strategies for CTC detection are in active development [17].

## TOOLS FOR ISOLATION AND MOLECULAR CHARACTERIZATION OF DTCs

### Enrichment, detection and isolation

**Figure 1:**
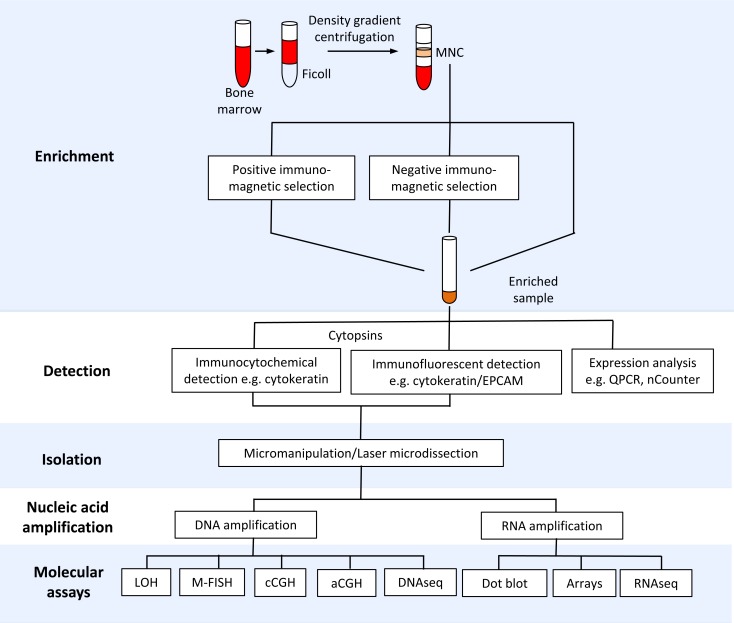
Tools for isolation and molecular profiling of disseminated tumor cells (DTCs) The schematic diagram shows bone marrow processing for enrichment, detection, isolation and downstream molecular profiling of DTCs. Abbreviations: MNC-mononuclear cells, QPCR-quantitative polymerase chain reaction, LOH-loss of heterozygosity (e.g. microsatellite and restriction fragment length polymorphism analysis), M-FISH-multiplex fluorescent in situ hybridization, cCGH-chromosome comparative genomic hybridization, aCGH-array comparative genomic hybridization.

**Figure 2:**
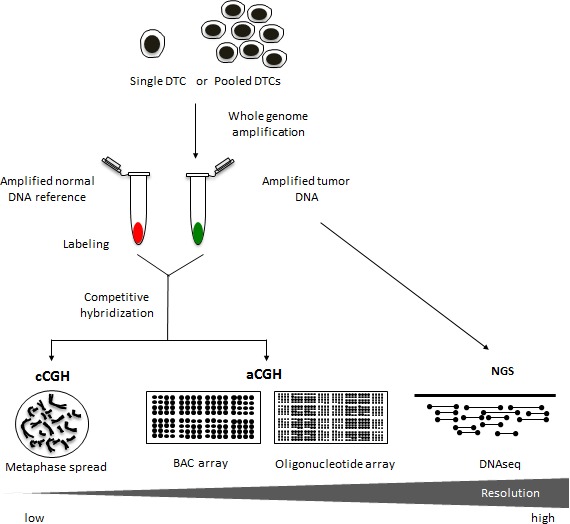
Copy number analysis using different genomic platforms Chromosome comparative genomic hybridization (cCGH) analysis has historically been a very valuable tool for detecting genomic copy number aberrations in DTCs. This approach, however, can be fairly labor intensive with limited resolution at approximately 5-20Mb [70]. Over the years, the switch from metaphase spreads to DNA microarrays has vastly improved the CGH method [71]. Array CGH (aCGH) platforms have been reported to achieve resolutions at around 0.1-5Mb for BAC arrays and >0.1Mb for oligonucleotide arrays [72-74]. The availability of standardized DNA microarrays along with a wide variety of bioinformatic tools has facilitated the streamlining of the aCGH procedure for genome-wide copy number analysis of tumor genomes. Moreover, recent advancements in DNA sequencing technology such as next generation sequencing (NGS) offer a resolution that is highly flexible ranging from single nucleotide variants to the identification of copy number alterations involving larger regions of the genome [40]. Copy number aberrations are typically extracted from NGS data by first dividing the reference genome into bins and counting the number of sequencing reads in each bin [41]. Using computational tools, copy number across the genome can then be inferred from the read counts [42].

Due to the high cellularity of the bone marrow, an enrichment step is most often required to facilitate the detection and isolation of rare DTCs. Methods for enrichment and detection of DTCs have been reviewed in detail [1, 18, 19]. Most enrichment strategies exploit physical (e.g., cell density) and biological properties (e.g., expression of epithelial markers) (Figure 1, Table 2 and Supplementary Table 1). For example, a majority of the studies discussed here have utilized density gradient centrifugation to separate the buffy coat, which contains mononuclear cells (MNC) and DTCs (Supplementary Table 1). This cell admixture can be subjected to further enrichment using immunomagnetic approaches involving iron beads coated with antibodies to cell surface markers. For example, positive and negative immunomagnetic selection methods have been utilized to enrich for cells expressing EPCAM or CD45 (leukocyte specific marker), respectively. DTCs in enriched samples can be detected via immunocytochemical or immunofluorescent assays to identify cells expressing epithelial markers such as cytokeratins or EPCAM (Supplementary Table 2). Enriched samples containing mostly marrow cells can be subjected to PCR-based expression profiling to detect tumor-specific transcripts [20, 21]. However, downstream molecular assays that are sensitive to the presence of marrow cells with normal diploid genomes (e.g., comparative genomic hybridization, see Figure 2) require highly pure DTCs. Techniques for complete isolation of DTCs include the preparation of cytospins from enriched samples and micromanipulation or laser microdissection of EPCAM- or cytokeratin-positive cells (Supplementary Table 2).

**Table 2:** Methods for enrichment, detection and isolation of disseminated tumor cells

Methods	Basis	Examples
**Enrichment**		
Density centrifugation	Physical properties	Ficoll-Hypaque density gradient, Percoll solution
Immunomagnetic beads	Cell surface markers	Positive selection using Anti-EPCAM; Negative selection using Anti-CD4S, -0061, -CD11b, -CD33, -CMS, -235a
**Detection**		
Immunocytochemical/Immunofluorescence	Epithelial markers	Detection of cytokeratin- and EPCAMopositive cells using fluorescent or chromogenic markers
**Isolation**		
Micromanipulation/Laser microdissection	Epithelial markers	Isolation of cytokeratin- and EPCAM-positive cells using fluorescent or chromogenic markers

### Nucleic acid isolation and amplification

The potential loss of nucleic acids during isolation has limited the utility of nucleic acid purification from single or small pools of cells prior to downstream molecular analysis. Alternatively, cell(s) can be lysed and the subsequent steps (e.g., nucleic acid amplification) can be performed within the whole cell lysates (Supplementary Table 3). Due to the limiting amount of nucleic acids from single DTCs, whole genome or whole transcriptome amplification is required to yield nano- to microgram quantities needed for high-throughput molecular assays. A unique approach that circumvents the need for nucleic acid amplification involves the *in vitro* propagation of DTCs [22, 23]. Examples of methodologies for single cell whole genome or whole transcriptome amplification are listed in Supplementary Table 3.

### Molecular assays

A wide variety of molecular techniques have helped elucidate novel insights into the molecular biology of DTCs (Table 1). Below, we provide brief overviews of the genomic DNA and expression profiling assays utilized in the studies discussed in this review.

**DNA assays.** The significance of genomic instability in cancer has encouraged attempts at documenting loss of heterozygosity (LOH) events in DTCs [24-26]. LOH can be detected as microsatellite instability [27] or as restriction fragment length polymorphisms (RFLP)[28], which are characterized by allelic loss of simple sequence repeats or restriction enzyme recognition sites, respectively. Among DTCs with no obvious copy number alterations, the detection of sub-chromosomal aberrations like LOH provided evidence for malignant origin [24-26].

A cytogenetic technique known as multiplex fluorescent *in situ* hybridization (M-FISH) has permitted karyotypic analysis in DTCs [22]. This method allows for the visualization of the entire genome using colored probes specific for each chromosome [29]. M-FISH facilitates the detection of chromosomal aberrations including euploidy, aneuploidy, and chromosome rearrangements.

The most widely used genomic profiling technology employed in copy number analysis of DTCs is chromosome comparative genomic hybridization (cCGH, or conventional CGH) [8, 22-26, 30-34]. In this method, tumor genomic DNA and normal reference DNA are differentially labeled with fluorescent dyes, and are co-hybridized to a metaphase spread [35]. The relative fluorescence intensities along the chromosomes in the metaphase spread reflect the copy number changes in the tumor genome. Despite its low resolution, cCGH has been a reliable tool in detecting chromosome losses, gains and amplifications. The development of array CGH, however, has addressed the issue of limited resolution of cCGH (Figure 2). Instead of a metaphase spread, tumor and normal DNA are co-hybridized to arrayed genomic probes consisting of bacterial artificial chromosome (BAC) clones [22, 32, 36] or short oligonucleotides [34, 37-39]. Finally, the development of NGS provides a highly sensitive means of detecting genomic variants including copy number aberrations in DTCs [37]. NGS involves the simultaneous sequencing of millions of DNA strands using multiplexing strategies [40]. The number of sequencing reads in specific regions of the genome is then used to estimate copy number [41, 42].

**RNA assays.** In addition to genomic DNA characterization, there have been efforts to study the transcriptome of DTCs using a variety of molecular techniques [20, 21, 30, 34, 43]. An example is dot blot hybridization, a technique capable of estimating the relative amount of RNA in a sample based on homology [44]. In this method, nucleic acids corresponding to genes of interest are blotted onto a membrane. The sample is then allowed to hybridize and the blotted sequences function as “probes” to capture homologous sequences. With the miniaturization of the fundamental chemistry of homologous hybridization, microarrays have superseded dot blot technique as one of the primary methods for expression analysis. Expression microarrays offer a major advancement in high throughput expression analysis by virtue of their design [45]. In contrast to traditional methods like QPCR, the arrayed DNA probes allow for the simultaneous detection and measurement of thousands of transcripts. A novel strategy for the direct measurement of transcript levels involves a digital bar coding system, also known as nCounter [20, 46]. Within such a system, color-coded molecular reporters are designed to hybridize to specific mRNA of interest and single-molecule imaging is used to detect and count the number of unique transcripts. Another approach for expression analysis called RNAseq makes use of NGS capabilities to sequence cDNA to estimate transcript abundance [47]. This technology, however, has yet to be applied to DTC transcriptome analysis.

## MOLECULAR CHARACTERIZATION OF DTCs

In the following sections, we review studies describing molecular characterization of DTCs. The studies have spanned a decade and half with the first genome-wide analysis reported in 1999 [24]. A majority of the studies focused on the characterization of DTCs from breast and prostate cancer patients (Table 1). Most involved copy number profiling of genomic DNA (13 of the 18), three involved RNA expression analysis, and two performed parallel DNA and RNA analyses (Table 1 and Supplementary Figure 1).

### DNA profiling

Klein and colleagues [24] were the first to demonstrate via cCGH, the presence of genome-wide copy number aberrations in cytokeratin-positive cells found in the bone marrow of cancer patients. First, a whole genome amplification method using ligation-mediated PCR (LM-PCR) was developed to accurately amplify genomic DNA of single DTCs. The amplification protocol was then applied to four individual DTCs from the bone marrow of a cancer patient with unknown primary lesion (CUP syndrome), and to cells isolated from liver metastasis of the same patient. The cCGH analysis revealed congruent patterns of genomic changes among the DTCs and the liver metastasis. DTCs showed a loss on chromosome 17p containing the *TP53* tumor suppressor gene and sequencing analysis revealed the inactivation of the remaining allele by a mutation. Further loss-of-heterozygosity (LOH) analysis revealed allelic losses in tumor suppressor genes, *APC* and *CDH1*.

In a follow-up study [30], bone marrow samples were collected from breast, prostate and gastrointestinal cancer patients with either clinically manifest metastasis (M1) or minimal residual cancer (M0). Two or more cytokeratin-positive cells from 71 samples were isolated and subjected to whole genome amplification (via LM-PCR) and cCGH analysis. Cells from 29 samples revealed normal genomes while the other 42 samples containing 115 cells harbored observable genomic alterations. M1 patients displayed significantly more aberrations per cell compared to M0 patients. Furthermore, cluster analysis demonstrated that M0 DTCs were much more heterogeneous than M1 DTCs. Cells isolated from lymph node, and DTCs from serial bone marrow samples from the same M0 patient shared almost no aberrations. Additionally, single stranded polymorphism analysis conducted on the 115 cells showed mutations in the *TP53* gene in 19 cells, while the rest did not carry any detectable mutations.

In an effort to understand the dynamics of tumor shedding, Schmidt-Kittler et al [26] analyzed cCGH profiles from single DTCs isolated from 83 breast cancer patients (M0=58 and M1=25) and compared them to primary tumors. Interestingly, the majority of M0 DTCs analyzed possessed normal profiles while M1 DTCs were found to be predominantly aberrant. Additionally, certain genomic aberrations were much more frequently shared among M1 DTCs, while very few aberrations were common among M0 DTCs. Further comparative analyses revealed significantly fewer aberrations in M0 DTCs as compared with matched primary tumors. Moreover, the pattern of genomic aberrations in primary tumors was not observed in matched M0 DTCs. While most M0 DTCs exhibited aneuploidy, M1 DTCs exhibited different types of aberrations including chromosome arm gains and losses, and focal amplifications. LOH analysis on M0 DTCs with normal genomes detected sub-chromosomal deletions indicating their malignant origin. Similar LOH patterns were observed in a subset of matched M0 DTCs and their corresponding primary tumors.

Kraus and colleagues applied an alternative strategy for characterization of DTCs through the establishment of two unique bone marrow cell lines (PC-E1 and PC-R1) derived from 2 M0 prostate cancer patients [22]. Although M-FISH karyotyping analysis of cultured DTCs detected increased ploidy, copy number aberrations and structural rearrangements in these cells, none were common to both cell lines. Shared aberrations between DTCs and the matched primary tumor as revealed by cCGH suggested a clonal relationship. Additionally, high-resolution copy number analysis via aCGH revealed aberrations common to both cells lines that were not detected using cCGH analysis. Interestingly, the chromosomal abnormalities observed in cultured DTCs have been previously documented in prostate cancers, e.g., 8p deletion and 8q gain. Also, the improved resolution using microarrays allowed for the detection of focal gains and deletions in these *in vitro* models.

Using a similar strategy, Gangnus and colleagues [23] established cell line models of DTCs from bone marrow of 5 M0 stage breast cancer patients. *In vitro* cell culturing allowed for the genetic analysis of viable and proliferative DTCs. Examination of the proliferation rates of cytokeratin-positive cells (putative DTCs) revealed no correlation with the proliferative potential of matched primary tumors as determined by Ki-67 staining. In contrast to previous findings by Schmidt-Kittler et al [26], cCGH analysis demonstrated that a majority of M0 DTCs exhibited genomic aberrations. Interestingly, some cases showed more genomic imbalances in DTCs in comparison to their primary tumor. The little resemblance of genomic profiles between DTCs and their matched primary tumor samples provided further evidence of early dissemination and independent evolution of DTCs.

The detection of DTCs with normal karyotypes [26] prompted investigation into whether these cytokeratin-positive cells were truly tumor cells. To provide evidence of malignant phenotype, Schardt et al [25] subjected 97 cells from 47 breast cancer patients to LOH analysis to detect sub-chromosomal aberrations. Included in the analysis were M0 cells with normal genomes as well as M0 and M1 cells with aberrant genomes as defined by previous cCGH analyses [26]. All three types of cells showed significantly higher frequency of LOH than control cells composed of normal blood cells from age-matched cancer patients. M0 cells with normal cCGH profiles exhibited losses in genomic regions containing *CDH1* (E-cadherin) and *CTNNB1* (β-catenin), suggesting that they are indeed tumor cells. Comparative analysis with matched primary tumors also revealed shared allelic losses. Of 16 matched cases analyzed, 9 showed at least one area within the matched tumor that shared at least one LOH with its corresponding DTC. Finally, a QPCR-based assay revealed significantly higher incidence of *HER2* gene amplification in M1 cells compared to M0 cells, suggesting that this aberration is acquired late in the diseases process. Intriguingly, comparison of the HER2 status between DTCs and matched primary tumors revealed poor congruence with only 1 of the 27 matched cases being concordant.

Single DTCs from 30 patients with esophageal cancer were subjected to copy number analysis by cCGH [33]. Results revealed numerous aberrations in these cells, which included highly recurrent gains on 17q12-21 containing the *HER2* locus. A comparison of genomic profiles between DTCs and single tumor cells from lymph node metastasis of the same patients showed almost no common alterations. For example, tumor cells from the lymph node exhibited gains on 7q and 10q, and losses on 5q; these alterations, however, were absent in DTCs. Despite this genetic divergence, gain of 17q12-21 (*HER2*) was observed at similar frequencies between the two groups. Quantitative assessment via QPCR analysis confirmed amplification of *HER2* in a subset of DTCs with gains on 17q12-21. Interestingly, discordant copy number aberrations including *HER2* status were observed between DTCs and matched primary tumors. Survival analysis demonstrated that patients whose DTCs had amplified *HER2* had significantly reduced survival compared to those without *HER2* amplification. In contrast, *HER2* status of matched primary tumors was not associated with increased risk of death.

To address the issue of limited resolution of cCGH, a protocol was optimized using a custom 3K BAC array (1Mb resolution) to analyze copy number in single cells [32]. Initial performance testing demonstrated that the BAC array outperformed other higher resolution arrays (i.e., 19K and 244K arrays) in detecting small gains and losses. Preclinical studies using single cultured cells attested to the reproducibility of the BAC aCGH assay. Analysis of three single DTCs from a breast cancer patient using the optimized aCGH protocol showed high correlation with cCGH results. As expected, additional aberrations that were not detected using cCGH analysis were apparent in the aCGH data. QPCR validation of genomic aberrations uncovered from aCGH analysis confirmed the copy number amplification of *AKAP3* and *AKAP6*.

The inconsistency between the high incidence of DTCs and the low rates of biochemical recurrence and metastasis among prostate cancer patients raised questions of whether DTCs were indeed tumor cells or merely non-malignant epithelial cells residing in the bone marrow [36]. Although aCGH analyses had previously been applied to isolated single DTCs [32] or to cultured DTCs [22], Holcomb and colleagues were the first to apply this method to small pools of cells. In this study, pools of 10-20 putative DTCs from 11 metastatic patients and 48 patients with localized disease were subjected to BAC aCGH. Copy number analysis revealed that DTCs from metastatic patients harbored genomic alterations previously reported in prostate cancers. These include loss on 8p, gain on 8q, and focal amplification on the X chromosome containing the androgen receptor (*AR*) gene. Although fewer and less striking genomic aberrations were found in DTCs from patients with localized disease, similarities with matched primary tumors provided strong evidence that these were truly tumor cells.

In a related study involving non-metastatic prostate cancer patients, DTCs were isolated before surgery (n=14 patients) and during clinical follow-up (n=23 patients) [8]. Isolated cells were subjected to cCGH analysis to examine genomic changes over time. In addition, DTCs extracted from patients who experienced biochemical relapse (n=14), and from patients with bone metastasis (n=12) were also examined. Copy number analysis revealed that DTCs from all groups displayed genomic alterations with very few cells showing normal profiles. There were no changes observed in the number of genomic aberrations per cell before and after surgery, and during biochemical relapse. DTCs from metastatic patients, however, had significantly more aberrations compared to the other groups, including matched primary tumors subjected to parallel cCGH analysis. Interestingly, DTCs from non-metastatic patients displayed heterogeneous aberrations while those from metastatic patients harbored shared aberrations, such as an 8p loss and 8q gain. Clustering analysis showed divergence in genomic profiles between primary tumors and the corresponding DTCs from metastatic patients, suggesting that the accumulated aberrations reflected in DTCs may have occurred late in the disease process.

A workflow for single cell aCGH analysis was developed using high-resolution oligonucleotide microarrays to allow in-depth assessment of genomic aberrations in single DTCs [38]. Initial experiments to determine the limits of resolution of the aCGH platform were performed on single cultured cell lines, CTCs from blood and normal blood cells. The optimized protocol was then applied to single DTCs from 3 early and 4 metastatic breast cancer patients. Copy number analysis showed genomic aberrations in DTCs that were also frequently seen in corresponding primary breast tumors. In addition, cluster analysis showed that genomic profiles of DTCs from the same patients were more similar to each other than DTCs from other patients. Interestingly, primary tumors shared similarities with corresponding DTCs at the time of diagnosis and at three-year relapse-free follow-up. This observation suggested the late dissemination of genomically advanced cells from the primary tumor, and the capacity of DTCs to stay dormant for extended periods of time.

A two-step assay involving whole genome amplification and oligonucleotide microarray analysis was optimized to analyze copy number changes in single DTCs [39]. Preclinical testing using cancer cell lines demonstrated reproducibility as well as sensitivity of the assay to detect cell-to-cell heterogeneity and to correctly quantify gene amplification. The workflow was then applied to single DTCs isolated from 4 serial bone marrow samples from a metastatic breast cancer patient receiving chemotherapy. The primary tumor and lymph node metastasis were also subjected to the same analysis. Copy number data generated from single DTCs and from corresponding tumor tissues revealed shared aberrations, e.g., gain in 8q (including the *MYC* oncogene) and loss in 13q (including the *RB1* tumor suppressor). Clustering analysis indicated that DTC copy number profiles were more similar to that of the lymph node metastasis than to the primary tumor. Detailed analysis of copy number profiles of single DTCs revealed substantial heterogeneity, but also uncovered the existence of DTC subclones that closely resembled the primary tumor and lymph node metastasis.

The first application of NGS in DTC analysis was performed to detect copy number aberrations and copy neutral loss of heterozygosity (i.e., LOH but with no net change in copy number) [37]. DNA from single DTCs from 2 early stage breast cancer patients was amplified and subjected to whole genome sequencing. A 2-3x depth of coverage was achieved allowing copy number assessment at ~50kb resolution. High concordance between the NGS-derived copy number profiles of single DTCs and the existing CGH data for corresponding primary tumors suggested a clonal relationship. In one of the DTC-primary tumor pairs, frequent chromosomal rearrangements (chromothripsis) localized on chromosome 2 were observed. Divergent sub-clonal changes were also noted between the primary tumor and single DTCs, indicating genetic progression. For example, one of the primary tumors exhibited a deletion in chromosome 13 while the corresponding DTC showed a copy neutral LOH in the same region.

### RNA profiling

To determine associations between gene expression and resistance to chemotherapy, microarray profiling was done on bone marrow samples enriched for EPCAM-expressing cells from 23 early stage breast cancer patients who received neoadjuvant chemotherapy [21]. Unsupervised hierarchical clustering revealed two distinct clusters: one with notable expression of transcripts associated with tumor invasion and metastasis (e.g., *TWIST1*), and the other cluster showing an over-representation of transcripts encoding ribosomal proteins, translational initiation factors, and genes involved in cell cycle and DNA repair. Differential expression analysis of EPCAM-enriched *vs*. whole bone marrow samples from the same patients revealed a DTC gene expression signature which included *TWIST1*. To validate the DTC signature, bone marrow samples from an independent set of 30 patients were interrogated via QPCR analysis. Fifteen patients had progressed or developed metastatic disease while the other half had stable disease for a year. Among the transcripts in the DTC signature, only *TWIST1* expression was significantly associated with early tumor relapse.

A gene expression-based approach involving the nCounter (Nanostring) system was developed for detection of DTCs in enriched bone marrow samples [20]. Initial testing using spiked cancer cells in healthy bone marrow samples demonstrated high concordance of nCounter with the “gold standard” QPCR assay. Subsequently, a panel of 38 transcripts was chosen for specific detection of DTCs following analysis of historical microarray data from primary breast tumors and bone marrow samples. Clinical application of the standardized method on bone marrow samples from 20 early stage breast cancer patients showed that only 20 of the 38 transcripts were detectable. Intriguingly, genes commonly used for DTC isolation such as *KRT19* and *EPCAM* were infrequently expressed, detected in 0% and 20% of the samples, respectively. Significant differential expression of *SNAIL2* and *LAMB1* was observed between patients who experienced a recurrence and those who did not. Candidate therapeutic targets, such as the *PTCH1* gene of the Hedgehog pathway, were expressed in patients who developed metastatic disease. In addition, discordance between primary tumor HER2 status and *ERBB2* expression in the bone marrow was commonly observed.

Chery and colleagues [43] performed oligonucleotide microarray analysis following whole transcriptome amplification to analyze gene expression profiles in single prostate DTCs. Of the 85 cells successfully profiled, 41 were deemed to be hematopoietic cells because of the high expression of an erythroid progenitor-like signature, and were excluded from the analysis. Clustering analysis of the remaining 44 cells—7 from 4 patients with no evidence of disease and 37 from 6 advanced cancer patients— revealed intra- and inter-patient heterogeneity. Furthermore, DTCs from advanced cancer patients clustered into two separate groups, with one closely resembling the expression profiles of DTCs from patients with no evidence of disease. Pathway analysis featured the enrichment of genes involved in the p38 stress response pathway, suggesting its role in regulating tumor latency that is consistent with the dormant nature of DTCs from patients in remission [48].

### Parallel DNA and RNA profiling

Klein et al [31] performed parallel copy number and transcriptome analysis in three single DTCs from patients with cervical, lung and breast cancer. CGH analysis performed on isolated single cells detected chromosomal aberrations consistent with malignant origin. Gene expression analysis via dot-blot hybridization revealed that the cervical DTC expressed positive regulators of cell cycle progression, while the lung DTC expressed genes involved in extracellular matrix degradation and systemic spread. The breast DTC showed expression of genes important for replication and cell cycle inhibition. Noteworthy was the high expression of EMMPRIN mRNA and protein observed in the majority of DTCs from additional prostate, breast and lung cancer patients studied. The expression of EMMPRIN in DTCs suggests its potential involvement in tumor invasion during early stages of metastatic spread.

Putative EPCAM-positive DTCs from 65 non-metastatic prostate cancer patients were subjected to targeted expression profiling of 17 genes [34]. The panel included epithelial, prostate-specific, tumor-specific, and hematopoietic transcripts to distinguish DTCs from normal bone marrow cells. EPCAM-positive cells from bone marrow of 10 healthy controls and 2 metastatic prostate cancer patients were also profiled. Expression analysis showed higher detection rates of *EPCAM* and *KRT* transcripts in single cells from non-metastatic prostate cancer patients than in the controls. Surprisingly, the *KLK3* gene, which encodes the prostate specific antigen, was rarely detected in prostate DTCs. In addition, the detection rates for hematopoietic (e.g., *PTPRC* or CD45), and erythroid (e.g., *HBA2*) transcripts were similar between the prostate DTCs and controls cells. Clustering analysis did not clearly separate the three groups from each other (control cells *vs*. DTCs from non-metastatic *vs*. DTCs from metastatic patients). Most of the single cells from the same patient did not cluster together suggesting intra-patient heterogeneity. Moreover, parallel CGH analysis showed that a majority of the cells did not possess genomic aberrations, and single cells harboring genomic aberrations were likely to express *KLK3* and epithelial transcripts. Notable was the detection of copy number alterations in some cells expressing erythroid and hematopoietic transcripts.

## BIOLOGICAL AND CLINICAL IMPLICATIONS OF DTC PROFILING

### Genomic instability

Genomic instability is a well-described hallmark of most cancers characterized by increased levels of genetic alterations involving complete or partial chromosomal loss and/or gain. It is therefore remarkable that numerous studies have shown that a majority of DTCs from non-metastatic patients exhibit very little instability. Those with aberrant profiles possessed fewer alterations in comparison to DTCs from metastatic patients, and displayed a repertoire of genomic aberrations that were infrequent in primary tumors of the same cancer type. Moreover, the common occurrence of aneuploidy relative to chromosome breaks suggested that dissemination occurred early before telomere crisis. In addition, these cells showed high cell-to-cell heterogeneity, whereas those from metastatic patients were more homogeneous, displaying similar patterns of genomic aberrations.

### Tumor heterogeneity

The significant genetic differences observed between DTCs and corresponding primary tumors have raised questions on whether the latter could be used as surrogates for the genetic makeup of existing metastatic cancer [49]. DTCs, on the other hand, can persist long after the primary tumor has been surgically removed, and therefore could potentially reflect the current status of the underlying disease [26, 50]. Unique information from real-time genomic analysis of DTCs may guide clinical decisions regarding which systemic therapy would be most effective for a patient. For example, HER2 targeted therapy may be administered to HER2-negative patients who harbor DTCs that are HER2-positive. Furthermore, serial analysis of DTCs before, during and after systemic therapy may help monitor changes in disease and assist in evaluating risk for local recurrence and/or distant metastasis.

### Tumor dormancy

An important question in metastasis research pertains to tumor cell dormancy, including whether and how DTCs can remain dormant over long periods prior to resumption of proliferative activity. Various studies have shown that DTCs can remain quiescent in the bone marrow for extended periods of time (reviewed in ref [51]). Attempts to culture DTCs have demonstrated their ability to proliferate, but with a very limited capacity [23, 52]. In addition, only a small subset of DTCs expressed the proliferation marker Ki-67, indicating that most cells were in a dormant state [53]. Factors involved in breaking dormancy leading to the proliferation and the formation of metastatic lesions are currently being investigated [51]. New treatment modalities that preserve DTCs in a dormant state may be an effective approach to prevent metastatic spread [1].

### Cancer stem cells

The cancer stem cell theory proposes that tumors arise from a unique subset of cells capable of self-renewal and multi-lineage differentiation [54]. It is hypothesized that a few cells within the DTC subpopulation possess stem cell-like properties. Consistent with a cancer stem cell phenotype, studies have shown that DTCs show resistance to chemotherapy [55-57] and display high expression of the stem cell marker, CD44 [58]. However, existing concerns regarding the robustness and specificity of available cancer stem cell markers [59] underscore a need for further studies to unequivocally demonstrate stem cell-like properties of DTCs.

### Epithelial-to-mesenchymal transition

During dissemination and migration, tumor cells may undergo a process called epithelial-to-mesenchymal transition (EMT), which results in the down-regulation of epithelial markers like cytokeratin and EPCAM [60]. Methods relying on epithelial markers may fail to detect DTCs undergoing EMT, often leading to false-negative findings. Therefore, novel markers that can facilitate the detection of this subpopulation of DTCs are needed. For example, increased expression of EMT-related markers such as *TWIST1*, *SNAIL1*, and *LAMB1* has been observed in DTCs [20, 21]. Whether these genes can serve as complementary markers to the current epithelial-based approaches requires more investigation [60]. In addition, the clinical significance of mesenchymal DTCs has yet to be demonstrated.

## EVOLUTIONARY MODELS FOR TUMOR PROGRESSION

**Figure 3:**
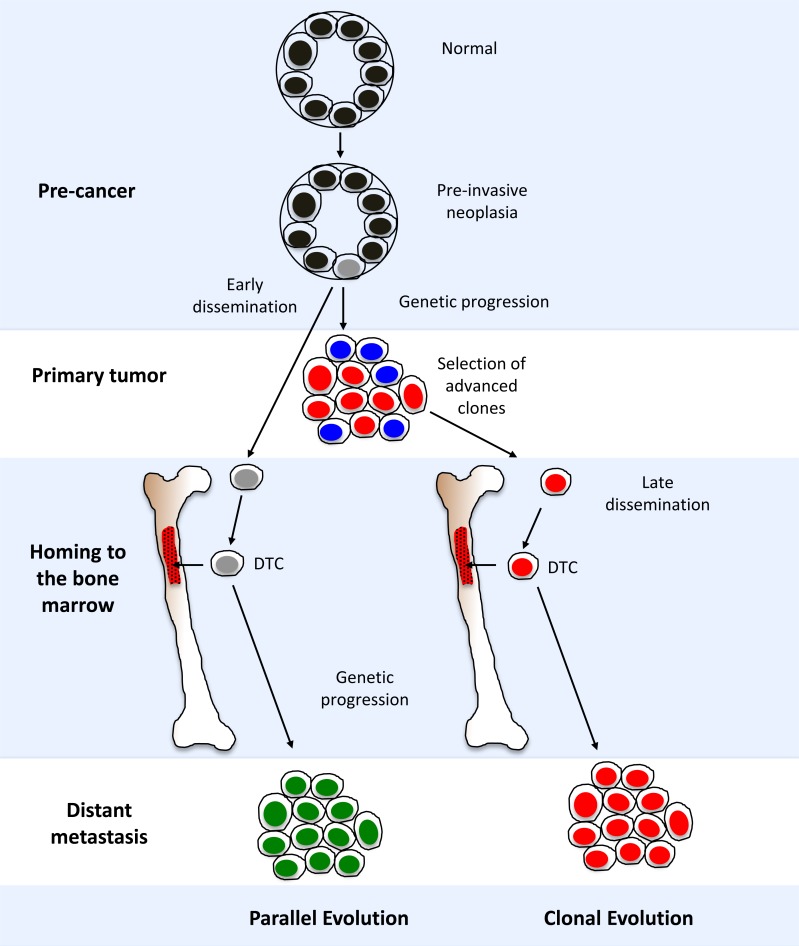
Models for disseminated tumor cell (DTC) evolution and cancer progression A diagrammatic representation of “parallel” and “clonal” evolutionary models. See section “Evolutionary models for tumor progression” in the main text for in-depth discussion.

Genomic analyses of DTCs and their corresponding primary tumors from non-metastatic patients have challenged the widely accepted ‘clonal evolution’ model for tumor progression (Figure 3). This model posits the selection of a subpopulation of metastasis-capable clones within the primary tumor, leading to hematogenous spread and localization in the bone marrow [61]. As direct descendants of primary tumor cells, DTCs inherit the same genomic alterations, plus additional events enabling or accompanying metastasis. However, studies have shown that matched primary and metastatic tumors can harbor many genetic differences, indicating additional complexity in this relationship [49, 62, 63].

An alternative ‘parallel evolution’ model proposes that DTCs disseminate early from the primary tumor, and acquire genomic aberrations independent of the primary tumor. The genomic profiles of DTCs and the primary tumor may therefore show little resemblance. For example, the detection of HER-positive DTCs from HER2-negative primary tumors suggests that *HER2* amplification may be actively selected for during dissemination and tumor progression [20, 25, 33]. The differences observed through profiling may be a manifestation of the genotypic or phenotypic changes when adapting to novel microenvironments [64]. Recent studies have also shown that dissimilarities between DTCs and corresponding primary tumors may be attributed to tumor heterogeneity found within solid tumors [37, 65, 66]. It is similarly hypothesized that DTCs may arise from rare subclones within the primary tumor whose characteristics do not represent the predominant genotype or phenotype [37].

Many of the studies in this review support the parallel evolution model [23, 26, 30, 33, 36, 67]. Some studies have demonstrated a clonal relationship between DTCs and the primary tumor by comparing sub-chromosomal aberrations (i.e., LOH) [25, 26] or copy number aberrations at higher levels of molecular resolution in single DTC subclones [39]. In addition, recent studies using high-resolution aCGH [22, 38] and NGS analysis [37] provide evidence in support of the clonal evolution model by demonstrating substantial similarities in genomic profiles between DTCs and matched primary tumors. It is possible that these competing models may both occur during the disease process [18]. A more recently proposed model, called “self-seeding”, describes an alternative route of tumor dissemination [68]. In this model, tumors can move bi-directionally, i.e., DTCs can return to the primary tumor of origin after dissemination. In-depth genomic analyses are needed to shed further light on the mechanisms involving self-seeding, selection of clones for dissemination, and the genetic relationship of DTCs with corresponding primary tumor and distant metastases.

## GENOMICS OF CTCs *VS*. DTCs

Much progress has been made recently towards genomic characterization of CTCs [69]. Despite a longer history of active study for DTCs as compared to CTCs, there are relatively few genomic studies of DTCs to date [69]. This may be attributed in part to the greater difficulty in obtaining bone marrow samples, which is a more invasive procedure compared to drawing blood. Perhaps more problematic is the inherent complexity of the bone marrow environment, since the diverse mature and progenitor populations from hematopoietic, stromal and other lineages can include cell types with potentially some phenotypic commonalities with malignant cells [43]. Improved technologies for DTC isolation away from confounding marrow populations will likely be an important advance to facilitate further detailed studies of DTCs. It is an exciting prospect that progress in both CTC and DTC genomics will eventually furnish two sources of information about cancer progression, and will allow direct comparison of these two processes.

## PERSPECTIVE AND FUTURE DIRECTIONS

Much remains unknown about the biology of DTCs. As with CTCs, the field of DTC genomic research is still in its infancy. Initial efforts towards characterization of DTCs have addressed the formidable technical challenges involved in detecting and characterizing these rare cells within the highly complex bone marrow environment. Subsequent improvements in rare-cell detection methods, combined with new techniques for characterization of limited amounts of tumor DNA, have facilitated progress in genome-wide copy number profiling of DTCs. Results from early genomic studies on DTCs have highlighted mechanisms involved in tumor cell dissemination and evolution.

Dissecting DTC biology may unlock key riddles about cancer progression. These include the temporal dynamics involved in DTC dissemination as early or late events, and the relationship of DTCs to primary tumors as direct progeny, echoes of shared ancestry, co-evolvers, and/or exemplars of heterogeneity. The relationship of DTCs to CTCs, bone metastasis and other distant metastasis remains unclear as well. DTCs can shed new light on the processes of genomic instability, tumor heterogeneity, dormancy, cancer stem cells, and epithelial-to-mesenchymal transition.

Future efforts towards comprehensive genomic analysis of DTCs will facilitate deeper understanding of DTC biology. Although first generation DTC testing did achieve a kind of proof of concept as a prognostic factor in early breast and other cancers, it never gained standard use as a clinical test. Genomic profiling of DTCs will likely lead to novel DTC-based biomarkers with pathogenetic and/or therapeutic relevance.

## SUPPLEMENTARY MATERIAL FIGURE AND TABLES


