# Supplementary material for: Approaches to isolation and molecular characterization of disseminated tumor cells
Source: Oncotarget. 2015 Sep 10;6(31):30715–29. doi: 10.18632/oncotarget.5568 (PMC4741563; doi:10.18632/oncotarget.5568)
Supplement: Supplementary file 1 [file oncotarget-06-30715-s001.pdf]

Approaches to isolation and molecular characterization of disseminated tumor cells

Supplementary Material

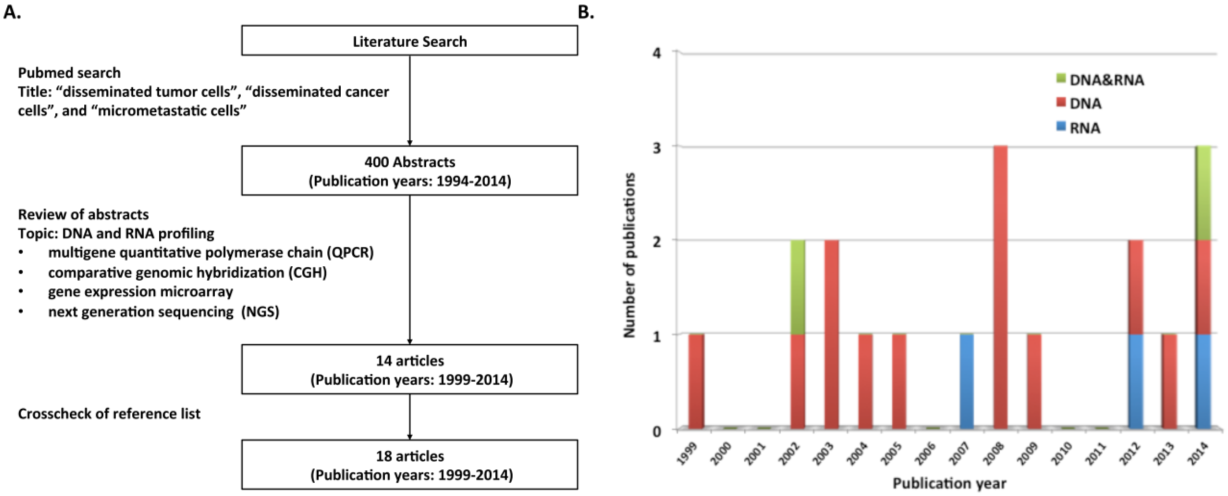

Supplementary Figure 1: Literature search methods and results. A) Flow chart of literature search; B) Number of scientific publications on DNA and RNA profiling of disseminated tumor cells.

Supplementary Table 1. Materials and methods for enrichment of disseminated tumor cells (DTCs). Abbreviation: MNC- mononuclear cells

Study	Enrichment approach	Type of enrichment technology	Reagents and antibodies	Phenotype for enrichment
**DNA profiling**				
Klein et al, 1999 PNAS	Density gradient MNC separation	MNC enrichment	Ficoll-Hypaque density gradient	Density > red blood cells
Klein et al, 2002 Lancet	Density gradient MNC separation	MNC enrichment	Ficoll-Hypaque density gradient	Density > red blood cells
Schmidt-Kittler et al, 2003 PNAS	Density gradient MNC separation	MNC enrichment	Ficoll-Hypaque density gradient	Density > red blood cells
Kraus et al, 2003 Genes Chromosomes Cancer	n.a.	n.a.	n.a.	n.a.
Gangnus et al, 2004 Clin Cancer Res	n.a.	n.a.	n.a.	n.a.
Schardt et al, 2005 Cancer Cell	Density gradient MNC separation	MNC enrichment	Ficoll-Hypaque density gradient	Density > red blood cells
Fuhrmann et al, 2008 Nucleic Acids Res	Density gradient MNC separation	MNC enrichment	Ficoll-Hypaque density gradient	Density > red blood cells
Stoecklein et al, 2008 Cancer Cell	Density gradient MNC separation	MNC enrichment	Ficoll-Hypaque density gradient	Density > red blood cells
Holcomb et al, 2008 Cancer Res	n.a.	n.a.	n.a.	EPCAM expression
Weckermann et al, 2009 J Clin Oncol	Density gradient MNC separation	MNC enrichment	Ficoll-Paque density gradient/Percoll 50% gradient	Density > red blood cells
Mathiesen et al, 2012 Int J Cancer	Density gradient MNC separation	MNC enrichment	Ficoll-Hypaque density gradient	Density > red blood cells
Moller et al, 2013 Front Oncol	Density gradient MNC separation	MNC enrichment	Ficoll-Hypaque density gradient	Density > red blood cells
Czyz et al, 2014 PLoS One	Density gradient MNC separation	MNC enrichment	Ficoll-Hypaque density gradient	Density > red blood cells
**RNA profiling**				
Watson et al, 2007 Clin Cancer Res	Density gradient MNC separation/Positive immunomagnetic	MNC enrichment/ Immunomagnetic bead	Ficoll-Hypaque density gradient/Anti-EPCAM immunomagnetic beads	Density > red blood cells/EPCAM expression
Siddappa et al, 2012 Breast Cancer Res Treat	Density gradient MNC separation	MNC enrichment	Ficoll-Hypaque density gradient	Density > red blood cells
Chery et al, 2014 Oncotarget	Density gradient MNC separation/Positive and negative immunomagnetic	MNC enrichment/ Immunomagnetic bead	Ficoll-Isopaque density gradient/Anti-EPCAM immunomagnetic beads/Anti-CD45,-CD61 immunomagnetic beads	Density > red blood cells/EPCAM expression
**DNA and RNA profiling**				
Klein et al, 2002 Nat Biotechnol	Density gradient MNC separation	MNC enrichment	Ficoll-Hypaque density gradient	Density > red blood cells
Guzvic et al, 2014 Cancer Res	Density gradient MNC separation/Negative immunomagnetic	MNC enrichment/Immunomagnetic bead	65% Percoll solution/Anti-CD11b,-CD33,-CD45,-235a immunomagnetic beads	Density > red blood cells/Hematopoietic marker-negative

Supplementary Table 2. Methods for detection and isolation of disseminated tumor cells. n.a.- not applicable.

Study	Detection approach	Isolation method	Definition of DTCs
**DNA profiling**			
Klein et al, 1999 PNAS	Immunofluorescent	Micromanipulation	Cytokeratin-positive
Klein et al, 2002 Lancet	Immunocytochemical	Micromanipulation	Cytokeratin-positive
Schmidt-Kittler et al, 2003 PNAS	Immunocytochemical	Micromanipulation	Cytokeratin-positive
Kraus et al, 2003 Genes Chromosomes Cancer	n.a.	n.a.	n.a.
Gangnus et al, 2004 Clin Cancer Res	Immunofluorescent	Laser Microdissection	Cytokeratin-positive
Schardt et al, 2005 Cancer Cell	Immunocytochemical	Micromanipulation	Cytokeratin-positive
Fuhrmann et al, 2008 Nucleic Acids Res	Immunofluorescent or immunohistochemical	Micromanipulation	Cytokeratin-positive
Stoecklein et al, 2008 Cancer Cell	Immunocytochemical	Micromanipulation	Cytokeratin-positive
Holcomb et al, 2008 Cancer Res	Immunofluorescent	Micromanipulation	EPCAM-positive
Weckermann et al, 2009 J Clin Oncol	Immunocytochemical	Laser microdissection and micromanipulation	Cytokeratin-positive
Mathiesen et al, 2012 Int J Cancer	Immunocytochemical	Laser microdissection and micromanipulation	Cytokeratin-positive
Moller et al, 2013 Front Oncol	Immunocytochemical	Micromanupilation	Cytokeratin-positive
Czyz et al, 2014 PLoS One	Immunocytochemical	Micromanupilation	Cytokeratin-positive
**RNA profiling**			
Watson et al, 2007 Clin Cancer Res	n.a.	n.a.	EPCAM-positive/Cytokeratin-positive
Siddappa et al, 2012 Breast Cancer Res Treat	Expression-based	n.a.	Expression of tumor-specific genes
Chery et al, 2014 Oncotarget	Immunofluorescent	Micromanupilation	EPCAM-positive, CD45-negative
**DNA and RNA profiling**			
Klein et al, 2002 Nat Biotechnol	Immunofluorescent	Micromanipulation	EPCAM-positive
Guzvic et al, 2014 Cancer Res	Immunofluorescent	Micromanupilation	EPCAM-positive

Supplementary Table 3. Methods for DNA and RNA profiling of disseminated tumor cells. RNA/DNA isolation, amplification strategies, platforms used, and numbers of patients successfully profiled in each scientific report. Abbreviations: WGA-whole genome amplification, WTA- whole transcriptome amplification, PCR- polymerase chain reaction, CGH-comparative genomic hybridization, UCSF-University of California San Francisco, BAC- bacterial artificial chromosome, QPCR- quantitative polymerase reaction, n.a.- not applicable

Study	DNA or RNA purification method	Amplification method (WGA or WTA)	Genomic analysis platforms	No. of cancer patients successfully profiled
**DNA profiling**				
Klein et al, 1999 PNAS	No purification/In lysate amplification	WGA: Ligation-mediated PCR	Metaphase CGH	1
Klein et al, 2002 Lancet	No purification/In lysate amplification	WGA: Ligation-mediated PCR	Metaphase CGH	71 samples
Schmidt-Kittler et al, 2003 PNAS	No purification/In lysate amplification	WGA: Ligation-mediated PCR	Metaphase CGH	83
Kraus et al, 2003 Genes Chromosomes Cancer	QIAamp DNA Minikit (QIAGEN)	n.a.	Metaphase CGH, UCSF BAC Array (HumArray1.14)	2
Gangnus et al, 2004 Clin Cancer Res	No purification/In lysate amplification	WGA: Ligation-mediated PCR	Metaphase CGH	5
Schardt et al, 2005 Cancer Cell	No purification/In lysate amplification	WGA: Ligation-mediated PCR	Metaphase CGH, SYBR Green QPCR	47
Fuhrmann et al, 2008 Nucleic Acids Res	No purification/In lysate amplification	WGA: Ligation-mediated PCR	Metaphase CGH, 3K BAC Array, SYBR Green QPCR	1
Stoecklein et al, 2008 Cancer Cell	No purification/In lysate amplification	WGA: Ligation-mediated PCR	Metaphase CGH, SYBR Green QPCR	30
Holcomb et al, 2008 Cancer Res	No purification/In lysate amplification	WGA: Ligation-mediated PCR	4K BAC Array	59
Weckermann et al, 2009 J Clin Oncol	No purification/In lysate amplification	WGA: Ligation-mediated PCR	Metaphase CGH	63
Mathiesen et al, 2012 Int J Cancer	No purification/In lysate amplification	WGA: GenomePlex Single Cell Whole Genome Amplification Kit (Sigma-Aldrich)	44K and 244K Human Genome CGH Microarray (Agilent)	7
Moller et al, 2013 Front Oncol	No purification/In lysate amplification	WGA: GenomePlex Single Cell Whole Genome Amplification Kit (Sigma-Aldrich)	244K Human Genome CGH Microarray (Agilent), HiSeq 200 (Illiumina)	2
Czyz et al, 2014 PLoS One	No purification/In lysate amplification	WGA: *Ampli1* WGA Kit (Silicon Biosystems)	SurePrint G3 Human CGH 4x180K Microarray (Agilent)	1
**RNA profiling**				
Watson et al, 2007 Clin Cancer Res	TRIzol Reagent (Invitrogen)	n.a.	Human Focus gene expression arrays (Affymetrix), Taqman QPCR	25
Siddappa et al, 2012 Breast Cancer Res Treat	TRIzol Reagent (Invitrogen)	n.a.	nCounter asssay (NanoString), Taqman and SYBR QPCR	20
Chery et al, 2014 Oncotarget	No purification/In lysate amplification	WTA: WT-Ovation One-Direct Amplification System (NuGEN)	Human 4x44K Microarray (Agilent)	10
**DNA and RNA profiling**				
Klein et al, 2002 Nat Biotechnol	No purification/In lysate amplification	WGA: Ligation-mediated PCR; WTA: Reverse transcrtion and PCR amplification	Metaphase CGH, Atlas human cancer 1.2 arrays (Clonetech Laboratories)	3
Guzvic et al, 2014 Cancer Res	No purification/In lysate amplification	WTA: oligo-dT reverse transcription and cDNA PCR amplification/WGA: *Ampli1* WGA Kit (Silicon Biosystems)	Targeted End-point PCR/Metaphase CGH/SurePrint G3 Human CGH 4x180K Microarray (Agilent)	67
